# Nanomedicines for Pulmonary Drug Delivery: Overcoming Barriers in the Treatment of Respiratory Infections and Lung Cancer

**DOI:** 10.3390/pharmaceutics16121584

**Published:** 2024-12-11

**Authors:** Raquel Fernández-García, Ana I. Fraguas-Sánchez

**Affiliations:** 1 School of Pharmacy, University of Nottingham, University Park, Nottingham NG7 2RD, UK; 2 Department of Pharmaceutics and Food Technology, School of Pharmacy, Complutense University, 28040 Madrid, Spain; 3 Institute of Industrial Pharmacy, Complutense University, 28040 Madrid, Spain

**Keywords:** pulmonary delivery, nanomedicine, infectious diseases, tuberculosis, cancer, dry powder inhalers, inhalable nanoparticles

## Abstract

The pulmonary route for drug administration has garnered a great deal of attention in therapeutics for treating respiratory disorders. It allows for the delivery of drugs directly to the lungs and, consequently, the maintenance of high concentrations at the action site and a reduction in systemic adverse effects compared to other routes, such as oral or intravenous. Nevertheless, the pulmonary administration of drugs is challenging, as the respiratory system tries to eliminate inhaled particles, being the main responsible mucociliary escalator. Nanomedicines represent a primary strategy to overcome the limitations of this route as they can be engineered to prolong pulmonary retention and avoid their clearance while reducing drug systemic distribution and, consequently, systemic adverse effects. This review analyses the use of pulmonary-administered nanomedicines to treat infectious diseases affecting the respiratory system and lung carcinoma, two pathologies that represent major health threats.

## 1. Introduction

The pulmonary route for drug administration has garnered a great deal of attention in therapeutics for both local drug action and systemic effect. This route is considered non-invasive, making its administration more comfortable for patients and improving treatment adherence [1,2,3]. On the one hand, the pulmonary route allows for the delivery of drugs directly to the lungs and, consequently, the maintenance of high concentrations at the action site and a reduction in systemic adverse effects compared to other routes, such as oral or intravenous. This is the preferred route for treating respiratory diseases such as asthma, chronic obstructive pulmonary disease (COPD), and idiopathic pulmonary fibrosis. In recent years, there has also been an increased interest in treating other pathologies affecting the respiratory system, such as pulmonary infections or lung carcinomas [4,5,6]. On the other hand, this route is also useful for systemic effects. The lungs have a large surface area (in the range of 70–140 m^2^ in adult humans) and a high vascularisation, which provides fast absorption and availability in the bloodstream and allows for a rapid onset of action. Moreover, it has a minimal metabolism compared with oral and other administration routes as it bypasses the hepatic first-pass effect [7,8]. 

**Figure 1:**
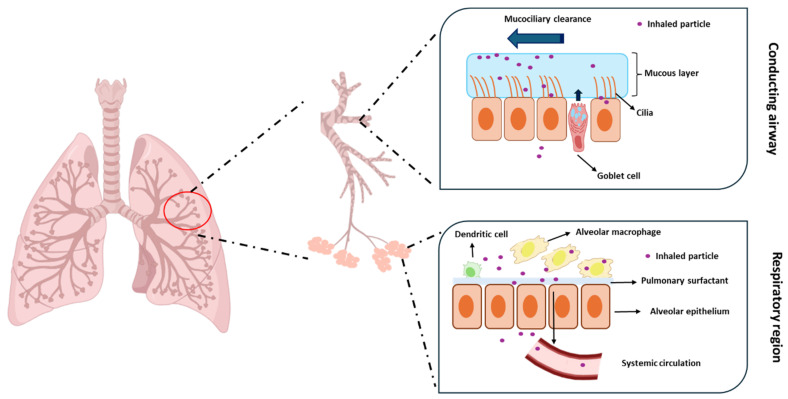
Schematic representation of the clearance of inhaled particles by the mucociliary escalator.

Nevertheless, the pulmonary administration of drugs is challenging. The respiratory system tries to maintain exogenous substances out of the lungs, removing or inactivating therapeutics after deposition. The conducting airways of the lungs are mainly layered by the mucociliary escalator, consisting of cilia embedded in a mucous layer [9,10,11]. The mucus is mainly secreted by goblet cells and consists of mucin, which is the principal structural and functional constituent, other smaller proteins, salt, and water [12,13]. The mucociliary escalator is one of the most important mechanisms responsible for removing inhaled substances. Mucins are glycoproteins with a high molecular weight crosslinked with cysteine residues [14]. They create a physical barrier that captures inhaled particles that are then removed by cilia and expelled from the respiratory system (Figure 1). The clearance of these particles occurs within 15 min to 2 h after inhalation, showing in healthy adults a mucociliary clearance in the range of 4–20 mm/min [15]. 

The immune system of the respiratory tract also plays an essential role in host defence and the removal of foreign particles. Alveolar macrophages are the most abundant cell type, accounting for more than 90% of the cells of this immune system. Inhaled foreign particles can be internalised by alveolar macrophages, transported to phagosomes, and degraded [16,17]. It should be mentioned that the alveolar space is covered by a layer of pulmonary surfactant comprising lipids and proteins that can interact with inhaled particles and facilitate their internalisation by alveolar macrophages and, consequently, their elimination [18]. 

**Figure 2:**
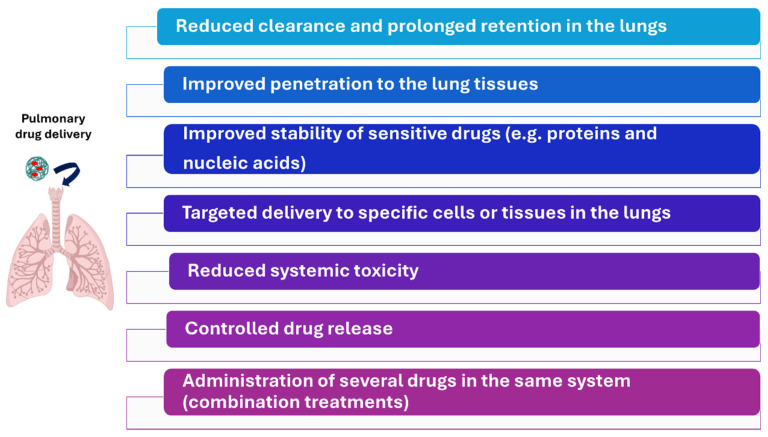
Advantages of nanomedicines over conventional formulations for pulmonary drug delivery.

Diverse physicochemical properties of the inhaled particles, such as particle size, surface charge and hygroscopicity, among others, condition the clearance and, consequently, the efficacy of this route [19]. It has been reported that particles above 1000 nm are easily entrapped in the mucous layer [11]. Particles with a positive surface charge are also quickly immobilised as mucus is negatively charged [20]. In this context, nanomedicines represent a promising strategy to overcome the limitations of the pulmonary route and improve its efficacy. Nanomedicines can be engineered to prolong pulmonary retention and avoid their clearance. They represent an excellent tool to improve the stability of drugs, protecting them from degradation (this is of particular interest for the delivery of proteins or nucleic acids); to release the drugs gradually, offering sustained therapeutic levels and reducing the frequency of administration; to access deeper regions of the respiratory system, as, due to their small particle size, they can penetrate deeper into the lungs; and to deliver drugs to specific cells of regions within the lung, among other advantages (Figure 2). Moreover, nanomedicines can be administered using inhalers and nebulisers [21,22,23]. This review analyses the use of pulmonary-administered nanomedicines to treat infectious diseases affecting the respiratory system and lung carcinoma. These are disorders where nanomedicines and the pulmonary route show a high utility. 

## 2. Nanomedicine in Pulmonary Drug Delivery

### 2.1. Types of Nanocarriers

Nanotechnology represents an excellent strategy for improving the efficacy and decreasing the toxicity of antimicrobials and antineoplastics [24,25,26]. Nanomedicines are also useful for overcoming the resistances developed by microorganisms and tumoral cells. These systems can evade efflux pumps, which actively expel conventional drugs from cells, reducing their effectiveness. Moreover, nanoparticles protect against enzymatic degradation, which can deactivate drugs. This latter mechanism is particularly important in microorganisms [27,28]. Nevertheless, it should be mentioned that pathogens, and cancer cells, can develop resistances to nanomedicines. For example, it has been demonstrated that bacteria induce the production of proteins like flagellin that trigger particle aggregation and make their internalisation difficult [29]. 

**Figure 3:**
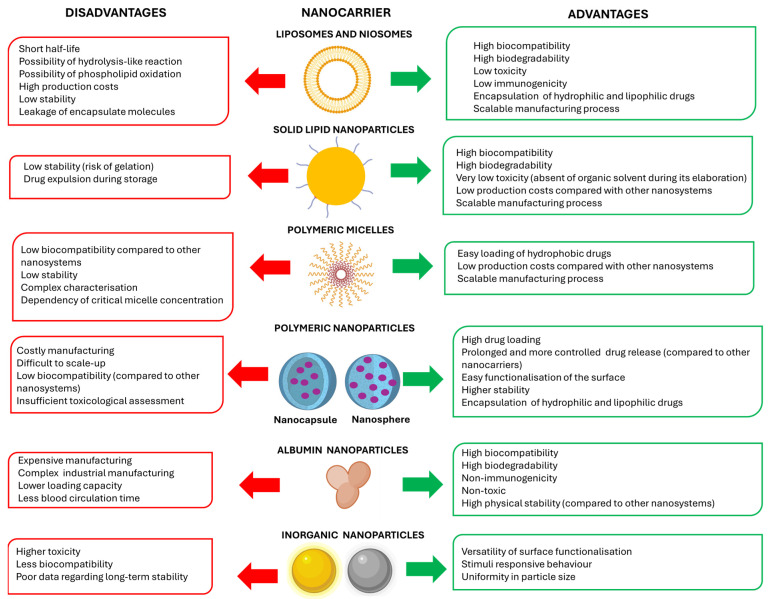
Advantages and disadvantages of each type of nanocarrier.

In general, nanoparticles can be divided into organic (including lipid, polymeric, and protein nanocarriers) and inorganic nanocarriers [30]. Among lipid nanocarriers, liposomes are particularly noteworthy [31]. They are spherical lipid nanovesicles consisting of an aqueous inner core surrounded by one or more bilayers of phospholipids. They are considered the most exploited nanocarrier for the delivery of both hydrophobic and hydrophilic molecules [32,33] due to several factors, including their high biocompatibility and easier manufacturing process at the industrial level compared to other types of nanomedicines [34,35] (Figure 3). Several liposomal formulations are currently approved for the delivery of antifungals (e.g., AmBisome^®^ or Arikayce^®^) and antineoplastics (e.g., Doxil^®^, Depocyt^®^, Mepact^®^ or Lipusu^®^), among other therapeutics [36]. Similarly to liposomes, niosomes have demonstrated a potential utility in drug delivery. They are self-assembled nanovesicles with an inner aqueous phase surrounded by a lipid bilayer composed of non-ionic surfactants, often combined with cholesterol [37,38]. Apart from liposome-type systems, solid lipid nanoparticles have also emerged as promising lipid drug delivery systems in the last decade due to their high biocompatibility, low toxicity, and industrially scalable manufacturing [39,40] (Figure 3). They are particularly interesting for the delivery of nucleic acids [41]. 

Polymeric nanocarriers are also valuable systems. Micelles are self-assembled nanoparticles of amphiphilic polymers with a hydrophobic core and a hydrophilic corona that have been widely investigated for the delivery of antineoplastics [42,43], with several formulations approved in the market (e.g., Genexol^®^ or Paclical^®^, both encapsulating paclitaxel) [44,45]. Polymeric nanoparticles offer different advantages, such as controlled drug release over extended periods, high versatility due to easily modifiable surfaces for controlling drug biodistribution, and superior drug encapsulation efficiency compared to other systems (Figure 3). They can be divided into nanocapsules (reservoir-type system), consisting of an inner core where the drug is usually dissolved surrounded by the polymeric layer, and nanospheres (matrix type system) composed of a continuous polymeric network in which the drug is entrapped. The latter are more commonly developed systems [46,47]. 

Albumin is a water-soluble globular protein that can bind many drugs by either covalent or non-covalent linkage, becoming an attractive carrier for drug delivery. It is a biocompatible, safe, and non-antigenic carrier, and albumin nanoparticles can be developed at the industrial level [48,49] (Figure 3). They are particularly interesting for administering antineoplastics as they tend to accumulate in the tumour area. Albumin nanoparticles bind to glycoprotein 60 receptors expressed on vascular endothelial cells of the tumours. Through these receptors, albumin nanoparticles are internalised to the tumour interstitium, where they bind to secreted acidic proteins rich in cysteine (SPARC) that are overexpressed in many tumours, such as lung, breast, and pancreatic carcinomas [50,51]. Abraxane^®^ and Fyaro^®^ are two formulations of albumin nanoparticles encapsulating paclitaxel and sirolimus, respectively, currently approved for treating several carcinomas [52]. 

Finally, inorganic nanoparticles such as gold nanoparticles, iron oxide nanoparticles and mesoporous silica nanoparticles have also emerged as promising nanoplatforms. They have shown great potential in diagnosis (with some formulations approved in the market for this purpose) and in drug delivery. Compared to organic nanosystems, inorganic nanoparticles exhibited a higher stability and ease surface of functionalisation, but a lower biodegradability and biocompatibility [53,54,55] (Figure 3). 

### 2.2. Impact of Physicochemical Properties of Nanoparticles on Pulmonary Drug Delivery

**Figure 4:**
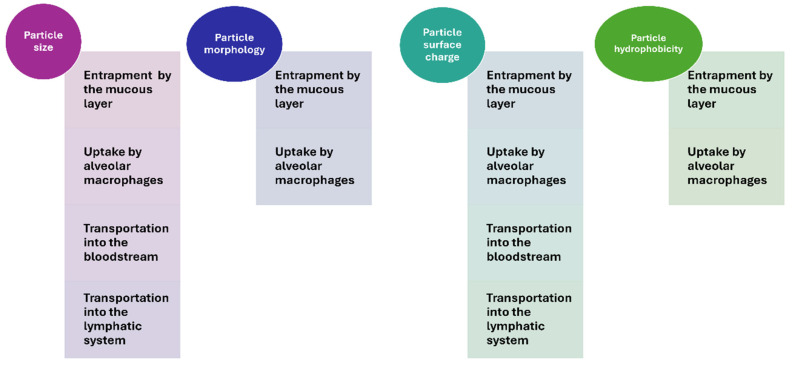
Impact of physicochemical properties of nanoparticles on pulmonary drug delivery.

Despite the considerable potential of the pulmonary administration route for treating respiratory diseases, the effective delivery of drugs by this route is challenged. Maintaining an aerodynamic particle size below 5 µm is essential for particles to effectively reach the mid and deep lung parenchyma. In this context, the use of nanocarriers is more than worthwhile. However, it should also be considered that particles smaller than 0.5 µm are usually prone to rapid exhalation [56], and consequently, developing pulmonary administered nanoformulations that can avoid rapid lung clearance is not easy. Particle size, particle morphology, surface charge, and hydrophobicity are critical parameters affecting the fate of pulmonary administered nanoparticles (Figure 4).

To optimise pulmonary drug delivery, innovative nanocarrier strategies, specifically ‘Trojan Horse microparticles’ or nano-in-microparticle, drug delivery systems have been developed. These advanced systems ingeniously integrate porous nanoparticles within microparticulate structures, typically ranging from 2 to 5 μm in diameter. By combining the targeted drug delivery capabilities of nanoparticles with the superior aerodynamic properties of microparticles, these hybrid systems offer a sophisticated approach to improving therapeutic effectiveness. The fabrication of these systems has been successfully achieved using different strategies, including the spray drying of supercritical CO_2_ [56].

#### 2.2.1. Influence of Particle Size

As aforementioned, the mucous layer of conducting airways offers a physical barrier that entraps inhaled particles as mucin glycoproteins create a crosslinked network acting like a filter. It has been reported that the upper airway region filters around 90% of inhaled particles [57]. Aerodynamic particle size is a critical parameter determining the retention of nanoparticles in the mucous layer and their mucociliary clearance. The larger the particle size, the higher the retention in the mucous layer. Schuster and collaborators demonstrated that polystyrene nanoparticles coated with polyethylene glycol (PEG) of 100 and 200 nm were not retained in the mucus. In comparison, particles of around 500 nm could not penetrate this layer efficiently, being entrapped [58]. Similar results were also found by Schenieider et al., who also reported that at least 70% of the administered dose of PEG-coated-polystyrene nanoparticles with a particle size around 300 nm were retained in the lung parenchyma 2 h after the administration [59]. The remaining 30% of the dose was removed from the respiratory tract. Finally, almost 90% of the administered 200 nm gold nanoparticles remain in the lung 2 h after their pulmonary administration [60]. 

Alveolar macrophages also play a significant role in the clearance of inhaled particles, phagocyting and degrading them, and particle size strongly influences macrophage phagocytosis. Macrophage uptake increases as particle size increases. Nanoparticles usually escape from alveolar macrophages due to their small particle size. Nevertheless, Chono and coworkers demonstrated that particle size was an influencing factor for liposome uptake. The phagocytosis of liposomal particles of 400 nm was two-fold higher than the uptake of liposomes of 100 nm [61]. 

Another aspect to be considered after the pulmonary administration of nanomedicines for treating respiratory disorders is the possible translocation of nanoparticles from the lungs into the circulatory or lymphatic systems. Very low amounts of intact nanoparticles can generally access the bloodstream or extrapulmonary organs after their pulmonary administration. However, some authors have reported this fact. For example, the capacity of PEG-poly(lactic-co-glycolic) acid (PLGA) micelles with a small particle size of around 28 nm to access the brain when administered intratracheally has been demonstrated in rats [62]. Kreling and collaborators demonstrated that particle size is a critical factor affecting nanoparticle transportation to the bloodstream. The translocation of 15 nm iridium nanoparticles was 1.5-fold higher than the transportation of 80 nm nanoparticles [63]. However, the amount of gold nanoparticles with a particle size of 20 and 200 nm was similar [60]. This suggests the influence of other properties of the formulations. 

Smaller nanoparticles are generally more easily transported to the lymphatic system by both cellular and non-cellular pathways. Choi et al. found that polystyrene nanoparticles with a particle size below 34 nm were rapidly transported from the alveolar luminal surface into the septal interstitium and then to the regional lymph nodes [64]. Similar results were also reported by Mohammad et al., who demonstrated that polystyrene nanoparticles of 50 nm had a higher overall lymph deposition (≈21%) compared to 100 and 250 nm nanoparticles (≈15 and 12.5%) 5 h after their administration [65]. 

Therefore, particle size is a critical parameter that conditions the efficacy of pulmonary-administered nanomedicines, as it influences their ability to access the mid-deep parenchyma and their ability to be retained in this region. Nanomedicines must be small enough to pass through the mucous layer of the respiratory conducting airways, penetrate the lung parenchyma, and avoid phagocytosis by the alveolar macrophages, but large enough to avoid rapid exhalation and translocation to the lymphatic and circulatory systems.

#### 2.2.2. Influence of Particle Morphology

Particle shape may also affect the fate of pulmonary administered nanoparticles, as it influences the uptake of alveolar macrophages. In general, it has been reported that this uptake is superior in spherical particles [66]. This aspect has been widely investigated and demonstrated in pulmonary administered microparticles [67,68], but less researched in nanoparticles. Several researchers have demonstrated that non-spherical (rod-shaped or pollen-shaped) microparticles show better flowability, aerosolisation, and deposition properties than spherical microparticles [68,69]. In this context, shifting from spherical to non-spherical nanoparticles may be a good technological strategy to decrease the macrophage clearance of inhaled particles.

#### 2.2.3. Influence of the Surface Charge

The surface charge of nanoparticles is another crucial factor that conditions the efficacy of pulmonary-administered nanoparticles. As aforementioned, the mucous layer is negatively charged due to the carboxyl and sulphate groups of the oligosaccharide chains [20], and positively charged nanoparticles are entrapped in this layer through electrostatic interactions, which may hinder their diffusion through the mucous layer and penetration to the lung parenchyma [70]. By contrast, neutral and negatively charged nanoparticles penetrate the mucous layer easily [11,71]. Dawson and collaborators found that neutral-charged polystyrene nanoparticles showed the highest mucus permeability [72]. In this context, coating nanoparticles with compounds that provide a neutral charge could be a good strategy to facilitate mucus penetration. For example, the coating of nanoparticles with dense PEG can bring the surface of the charge to neutral [68] and improve mucus penetration. Polystyrene nanoparticles coated with PEG exhibited superior mucus penetration. This penetration was 15- and 35-fold higher in 100 nm and 200 nm nanoparticles, respectively, compared to non-pegylated nanoparticles [58]. As positively charged inhaled nanoparticles are retained in the mucus, their mucociliary clearance is lower compared to non-positive nanoparticles. This could be useful when it is required to increase retention in the respiratory system. 

The surface charge of nanoparticles is also crucial for macrophage phagocytosis. It has been reported that positively charged nanoparticles are generally more easily phagocyted by macrophages due to their interaction with the negatively charged sialic acid of these cells [11]. Seydoux and collaborators reported that phagocytosis by alveolar macrophages of positively charged-polyvinyl alcohol (PVA)-gold nanoparticles was higher than negatively charged-PVA-gold particles [73]. Similar results were found in negatively and positively charged PLGA nanoparticles [74]. 

As positively charged nanoparticles tend to be retained in the mucous layer, the possibility of their translocation to the bloodstream is extremely low compared to negatively charged formulations. Kreyling et al. showed that around 5% of positively charged gold nanoparticles (≈3 nm) were detected in extrapulmonary organs 24 h after their intratracheal instillation, while negatively charged nanoparticles showed significantly lower translocation percentages (≈1%) [75]. Similar results were found in metallic oxide nanoparticles with negative and positive surface charges [76]. 

Surface charge can also influence the translocation of nanoparticles to the lymphatic system. Choi and collaborators demonstrated that it is critical in lymph translocation via the non-cellular pathway in particles below 34 nm. In this case, nanoparticles with a neutral or negative surface charge rapidly translocated from the lung to the mediastinal lymph nodes, while in positive nanoparticles, this translocation was inhibited [64]. The surface charge of nanoparticles did not seem to influence nanoparticles translocated via cellular pathways [77]. 

Therefore, the surface charge of nanoparticles is another key factor influencing the fate of pulmonary administered nanoparticles. Neutral and negatively charged nanoparticles show a lower interaction with the mucous layer and a higher penetration than positive nanoparticles, especially net-neutral formulation. Non-positive nanoparticles also demonstrated a lower uptake by alveolar macrophages and a lower translocation from the lungs to the blood circulation. 

#### 2.2.4. Influence of Surface Hydrophobicity

The surface hydrophobicity of the nanoparticles is another factor influencing the penetration of particles through the mucous layer. Large amounts of lipids can be absorbed or bound to the mucous, specifically to the mucin network of non-PTS (proline, threonine, and serine) regions. These lipids can interact with hydrophobic nanoparticles, favouring their retention. Hydrophilic nanoparticles can penetrate the mucous layer better, accessing the lung parenchyma. This also explains why coating the surface of the nanoparticles with PEG (a hydrophilic polymer) is a good strategy to improve their penetration [78]. However, not all PEG polymers are helpful for promoting penetration through the mucous layer. Wang and collaborators reported that the chain of PEG with high molecular weight can entangle with mucins and hamper nanoparticle penetration. However, low-molecular-weight PEG polymers avoid this problem [79]. In general, PEG polymers with a molecular weight in the range of 2 to 5 kDa are adequate to improve the penetration of inhaled nanoparticles through the mucous layer of conducting airways [80]. 

Incorporating PEG and other hydrophilic polymers on the surface of the particles also decreases their macrophage-mediated phagocytosis. For example, Murata and collaborators reported that incorporating PVA (PVA also provides a hydrophilic surface) to the surface of liposomes also decreases recognition and uptake by alveolar macrophages [81].

### 2.3. Impact of Patient-Specific Factors

Another key point to consider in the design of pulmonary administration medications is that respiratory function, as well as the mechanisms (e.g., mucociliary escalator, alveolar macrophage phagocytosis, and lung surfactant interaction) involved in the removal of inhaled particles, vary significantly across different patients, such as smokers or individuals with chronic lung diseases, affecting the success of inhaled therapies. 

#### 2.3.1. Smoking

Smoking significantly alters the respiratory system of patients, which in turn affects the outcome of inhaled medications [82]. One of the main consequences is a reduction in lung capacity and ventilation, which reduces the ability of the patient to inhale deeply. As a result, lower doses reach the lungs and the efficacy is reduced. Moreover, smoking leads to changes in the mucociliary escalator. Smoking reduces ciliary function and produces an exacerbation of mucus production [83]. The excess of mucus can retain more nanoparticles and hinder their diffusion to the lungs, decreasing their efficacy. Moreover, microorganisms can also be retained, increasing the risk of developing infections. 

#### 2.3.2. Chronic Obstructive Pulmonary Disease

Chronic Obstructive Pulmonary Disease (COPD) is characterised by a progressive reduction in lung function due to persistent airflow obstruction [84], which can influences the outcome of inhaled medications, including nanomedicines. In these patients, the secretion of mucus is exacerbated, producing a more viscous and harder-to-clear mucous layer that retains inhaled particles [85]. Moreover, the chronic inflammation of COPD increases the activity of alveolar macrophages, which can trigger an enhanced phagocytosis and reduces the dose that reaches the lungs. 

#### 2.3.3. Cystic Fibrosis

In patients with cystic fibrosis, a similar situation occurs. This disease also involves a chronic inflammation of the conducting airways, a decline in lung function and an excessive production of sticky mucus. Patients with cystic fibrosis experience multiple bacterial infections, and inhaled antibiotics represent a good treatment approach [86], including the administration of nanomedicines. However, it should be considered that this mucus is not only more viscous, but also enriched with DNA, proteins, and cellular debris, and creates a dense barrier that significantly hinders the penetration and distribution of nanoparticles to deeper areas of the respiratory system. 

#### 2.3.4. Asthma

Asthma is a prevalent condition characterised by the intermittent and reversible obstruction of respiratory airways, heightened sensitivity, and chronic inflammation of the respiratory system [87]. The pulmonary administration route is widely used for the treatment of asthma, providing effective and rapid relief by delivering therapeutic agents directly to the lungs. However, it is important to consider that the pathological damage associated with asthma, particularly chronic inflammation, can influence the accessibility and efficacy of inhaled formulations. Persistent airway inflammation leads to a thickening of the airway walls, increased mucus production, and altered epithelial permeability that may hinder the access of nanoparticles. 

#### 2.3.5. Lung Cancer

Inhaled nanomedicine holds significant potential for the delivery of anticancer drugs in lung cancer. However, this pathology induces changes in the respiratory system that can affect the efficacy of inhaled nanoformulations [88]. Firstly, the lung capacity of these patients may be reduced, which impacts the effectiveness of this therapy, as proper inhalation technique and adequate pulmonary function are essential. Moreover, tumours often induce chronic inflammation, leading to increased mucus production that retains nanoparticles and hinders their access to cancer cells. 

## 3. Pulmonary-Administered Nanomedicines to Combat Respiratory Tract Infections

A wide variety of bacteria, viruses, and fungi are the causative agents of respiratory infections. Respiratory tract infections occur in both the upper and lower regions of the respiratory system [89]. Upper respiratory tract infections are usually defined as self-limited irritation and inflammation of the upper conducting airways without evidence of pneumonia, and their complications are rare [90]. Nevertheless, lower respiratory infections are usually more severe and difficult to handle [91,92]. 

Lower respiratory infections are a major health problem. According to the World Health Organization (WHO), they represented the fifth leading cause of death globally in 2021 and the first cause in low-income countries. There are countless therapeutic options for their treatment, and in clinical practice, an empirical regimen of oral or parenteral antimicrobials is followed [93]. Nevertheless, systemic side effects and drug resistance remain considerable limitations in treating respiratory infections [94]. The administration of antimicrobials by the pulmonary route can overcome these challenges. Compared to oral or parenteral routes, pulmonary administration delivers drugs directly into the lungs, allowing for high therapeutic concentrations at the action site with lower doses and reducing the systemic exposure of antimicrobials and, consequently, systemic adverse effects [21,95]. In fact, due to the advantages of the pulmonary route in clinical practice, some intravenous formulations are repurposed for nebulisation [96]. Table 1 displays the available pulmonary formulations for treating bacterial and fungal infections.

Nevertheless, pulmonary drug delivery is challenging. Using nanocarriers may overcome the limitations of this route [97]. Moreover, nanoparticles can be engineered to overcome drug resistance. In fact, a liposomal formulation of amikacin for pulmonary administration (Arikayce^®^) is currently approved for treating nontuberculous mycobacterial lung disease in adults (Table 1) [98,99]. 

### 3.1. Bacterial Infections

Bacteria are the dominant causative agents of pulmonary infectious diseases [100]. They usually occur as a consequence of another respiratory condition, including viral infections, COPD, and cystic fibrosis [93]. These infections might be caused by different types of bacteria, including *Streptococcus pneumoniae*, *Legionella pneumophila*, *Mycoplasma pneumoniae*, *Chlamydia pneumoniae*, *Haemophilus influenzae*, and *Mycobacterium tuberculosis* [101]. Table 2 shows a summary of novel nanotechnology-based antibacterial therapy approaches.

#### 3.1.1. Tuberculosis

*M. tuberculosis* is the causative agent of tuberculosis (TB) [102]. While it is a preventable and treatable condition, TB continues to pose a significant global health challenge [103]. Each year, over 10 million individuals are diagnosed with the disease, predominantly in low-income nations [104]. Despite a substantial decline in TB-related mortality over the past three decades, it remains one of the leading causes of death worldwide, accounting for 1.6 million fatalities in 2021 [105,106].

The pharmacotherapy of TB has faced challenges due to the absence of an optimal treatment with favourable safety and pharmacokinetic profiles, despite the availability of these drugs for nearly 80 years. Additionally, drug-resistant TB is a major complication, contributing to over 10% of TB-related deaths [107,108]. The four cornerstone drugs used in anti-TB treatment (rifampicin, isoniazid, pyrazinamide, and ethambutol) exhibit diverse mechanisms of action and are administered in combination to prevent the development of resistance [109,110]. Alternative antibiotics, such as ciprofloxacin, levofloxacin, rifabutin, and linezolid can be employed as second- and third-line treatments [111].

Although TB primarily affects the lungs, current therapeutic options predominantly involve the systemic oral administration of antibiotics. This method may be nonspecific, potentially targeting other tissues and causing toxicity while also achieving inadequate drug concentrations in the lung [93]. Such issues can promote the emergence of resistance. Furthermore, TB treatment regimens are lengthy, typically lasting 6 to 9 months [112], which can lead to poor patient adherence, premature treatment discontinuation, relapse, and resistance development [95]. It is also important to note that *M. tuberculosis* is particularly challenging to eradicate. Alveolar macrophages play a crucial role as the first line of defence in the innate immune response against pulmonary infections; they phagocytise and destroy inhaled pathogens. However, *M. tuberculosis* evades macrophage responses by inhibiting phagosome maturation and preventing lysosome fusion, thereby transforming the hostile macrophage environment into one conducive to its survival and replication [113,114].

In this context, there is an urgent need for novel anti-TB therapies that enhance the tolerability and biodistribution of antimicrobials, promote efficacy with lower doses, increase patient adherence by shortening treatment durations, and reduce resistance emergence. The pulmonary route has yet to be fully utilised in clinical anti-TB therapies, but it offers potential advantages over systemic drugs, such as minimising systemic side effects and toxicity while achieving therapeutic concentrations at the target site with lower doses [115]. Nanomedicine has emerged as a promising approach for TB treatment, as it can be engineered to specifically target infected macrophages [116,117].

Various nanomedicine formulations have been developed to encapsulate anti-TB drugs, including liposomes, nanoparticles, polymeric micelles, and niosomes (Table 2) [107]. For instance, Ma et al. created mannose-modified solid lipid nanoparticles carrying a pH-sensitive isoniazid prodrug for pulmonary administration. The surface modification enhanced macrophage uptake significantly, achieving 97.2% compared to 42.4% for unmodified nanoparticles [118]. Viswanathan et al. developed inhalable liposomes containing liquorice extract, intended as a potential standalone treatment for TB or as an adjuvant to existing therapies. Their in vitro studies indicated favourable flow properties for inhalation, while in vivo studies showed that 46% of the administered drug reached the lungs, with 16% retained 24 h post-administration [119]. Silva et al. explored the use of antimicrobial peptides (AMPs), specifically LLKKK18, which has demonstrated effectiveness against *M. tuberculosis* and *Mycobacterium avium.* This AMP was encapsulated in self-assembling hyaluronic acid nanogels, enhancing stability, reducing cytotoxicity, and improving targeting to infection sites. In vivo studies indicated significantly reduced infection levels after just five doses [120]. Wu et al. introduced lung-targeted nanogel particles composed of genipin-crosslinked deacetylated chitosan loaded with isoniazid and rifampicin. These particles exhibited extended antibacterial activity due to sustained drug release, with a single pulmonary dose maintaining therapeutic concentrations of 40–60% in the lungs for 24 h while keeping levels in other organs below 5% [121]. Lastly, Scolari et al. designed nanoparticles made of sodium alginate coated with chitosan and Tween^®^ 80, encapsulating rifampicin and ascorbic acid, utilising a design-of-experiments approach to optimise the formulation. The addition of ascorbic acid, shown to enhance the efficacy of anti-TB drugs like isoniazid and rifampicin [122], resulted in improved activity against nine clinical strains of *M. tuberculosis.* The formulation also included sucrose or mannitol to facilitate easy redispersion and prevent particle aggregation during lyophilisation [123].

#### 3.1.2. Bacterial Pneumonia

Beyond TB, lower respiratory tract infections caused by other bacterial pathogens are also a significant issue, particularly in developing countries, which account for over 95% of global cases. It is estimated that around 4 million deaths occur annually due to these infections [124]. Bacterial pneumonia can be categorised into community-acquired pneumonia, primarily caused by *S. pneumoniae*, *M. pneumoniae*, *H. influenzae*, *C. pneumoniae*, and *L. pneumophila* (listed in order of frequency), and hospital-acquired pneumonia, which has a high mortality rate and is mainly associated with *Pseudomonas aeruginosa*, *Enterobacteriaceae*, and *Staphylococcus aureus* [125,126].

Timely administration of antibiotics is crucial to alleviate symptoms and reduced mortality associated with bacterial pneumonia. The choice of antibiotic depends on the causative agents and the severity of the illness. Commonly used classes of antibiotics include macrolides, doxycycline, β-lactams, aminoglycosides, fluoroquinolones, and carbapenems. In cases where the causative agent cannot be identified, empirical treatment with broad-spectrum antibiotics may be necessary, which can contribute to the development of resistant bacterial strains [95]. 

Inhaled therapies have also shown promise in treating bacterial pneumonia, and several nanoformulations for pulmonary drug delivery have been developed. For example, Arikayce^®^ is a liposomal formulation of amikacin approved for treating lung infections caused by the *M. avium* complex (Table 1). However, it is important to note that the excessive mucus production and inflammatory response in pneumonia patients can hinder the effectiveness of inhaled therapies [70].

Additionally, there are ongoing studies investigating nanomedicine-based treatments for the pulmonary administration of other bacterial infections (Table 2). One approach involves developing poly(2-ethyl-2-oxazoline) (PEtOx) nanoparticles designed to encapsulate ciprofloxacin, enhancing in vivo solubility and optimising lung delivery. These nanoparticles demonstrated sustained release for up to 7 days, indicating their potential as an alternative therapeutic option for lower respiratory tract infections [127]. AMPs have also gained attention as alternatives to traditional antibiotics for pulmonary infections [93]. One specific AMP, SET-M33, was incorporated into dextran-based nanoparticles targeting *P. aeruginosa* infections, resulting in significantly prolonged lung residence time (approximately 12-fold greater than the free peptide) while maintaining acceptable toxicity levels. This was achieved through non-covalent interactions under suitable pH conditions [128].

Derbali et al. compared poly(lactic) (PLA)-grafted-PEG nanoparticles with anionic and cationic liposomes to encapsulate levofloxacin, ultimately identifying anionic liposomes as the most promising formulation. These anionic liposomes exhibited a sustained release of levofloxacin over 72 h while preserving antibacterial activity against five strains of *P. aeruginosa.* Notably, despite their negative charge, the liposomes effectively interacted with *P. aeruginosa* membranes in a dose-dependent manner, demonstrating a favourable safety profile on A549 epithelial cells, even at high concentrations [129]. Another study investigated a liposomal formulation combining levofloxacin and lysozyme formulation for pulmonary delivery. This formulation was developed using a pH gradient method and spray dried with a lysozyme-containing solution. Efficacy against *S. aureus* was tested in rats, where the liposomes containing both levofloxacin and lysozyme significantly reduced microbial burden in the lungs, bronchoalveolar lavage fluid, and nasal fluid, with acute toxicity studies confirming the safety of the formulation [130]. Lastly, an innovative approach utilised liposomes containing colistin for treating *P. aeruginosa* infections. These liposomes were designed with sodium cholesteryl sulphate to enhance electrostatic interactions between the drug and lipids. In vivo studies demonstrated prolonged retention of colistin in the lungs while minimising transfer to the bloodstream and kidneys, leading to improved therapeutic efficacy in a murine model of pulmonary *P. aeruginosa* infection [131].

### 3.2. Fungal Infections

Respiratory fungal infections, similar to bacterial lung infections, pose a serious global health threat, causing approximately 1.5 million deaths annually [132]. Over recent decades, the incidence of these infections has risen, likely due to the growing population of immune-compromised individuals, resulting from factors such as organ transplants, cancer treatments, autoimmune diseases, acquired immunodeficiency syndrome (AIDS), and corticosteroid therapies, among others [133]. The primary pathogens behind fungal lung infections include *Aspergillus*, *Cryptococcus*, *Pneumocystis,* and *Candida albicans*, which account for over 90% of fungal infection-related deaths. Treatment strategies typically involve either oral or parenteral administration of broad-spectrum azoles (e.g., voriconazole or posaconazole) or the parenteral administration of amphotericin B or echinocandins (e.g., anidulafungin or caspofungin) [134,135]. Although amphotericin B is a key treatment for fungal infections, its parenteral application is often problematic for lung infections due to severe side effects, including kidney and liver toxicity [136,137]. Pulmonary drug delivery offers a promising alternative by reducing systemic exposure and toxicity. In some cases, intravenous formulations of antifungal are adapted for inhalation to take advantage of the localised effects of pulmonary delivery [96].

Nanotechnology has recently been explored to enhance the treatment of respiratory fungal infections through pulmonary delivery systems. Table 3 provides an overview of recent nanotechnology-based approaches. A novel approach by Kaur et al. involved using chitosan nanoparticles loaded with voriconazole, which were aerosolised into microdroplets for pulmonary administration. These nanoparticles showed effectiveness against *Aspergillus* species and improved lung retention in pharmacokinetic studies [138]. Another study by Ali et al. evaluated silver nanoparticles with *Artemisia sieberi* leaf extract in a mouse model of invasive pulmonary aspergillosis (IPA), which significantly reduced lung tissue damage by inhibiting fungal growth and biofilm formation, while also increasing antioxidant enzyme activity [139].

Further advancements include the development of a pulmonary dapsone delivery system to prevent *Pneumocystis carinii* pneumonia in immunocompromised patients. Dapsone was encapsulated in liposomes and formulated into a dry powder inhaler (DPI) with hydrolysed gelatin-based carriers for sustained release up to 16 h, following Higuchi’s release model [140]. Chitosan-stearic acid nanomicelles encapsulating amphotericin B have also been developed, showing enhanced antifungal activity and stability post-nebulisation [141]. Additionally, Nasr et al. reported that nebulised nanoemulsions successfully delivered amphotericin B to the peripheral respiratory airways. Formulations prepared with Clinoleic^®^ (an emulsion elaborated with olive oil, sodium oleate, egg phospholipids, soybean oil, and glycerol) showed a better performance than nanoemulsions based on Intralipid^®^ (fat emulsion prepared from egg phospholipids, soybean oil, and glycerol) with deposition percentages of around 80 and 57%, respectively [142]. Another study utilised ligand-coated liposomal aerosols with alveolar macrophage-targeting ligands for amphotericin B, which demonstrated efficient lung accumulation and prolonged retention in macrophages [143]. Finally, nanostructured aggregates containing mannitol and lecithin were created using ultra-rapid freezing for itraconazole delivery. This formulation displayed favourable aerodynamic properties, reaching the deepest lung regions and achieving systemic levels in mice [144]. 

**Table 2:** Novel formulations to treat bacterial pulmonary infections.

Formulation	Drugs	Mechanism of Action	Disease	Excipients	Comments	Reference
Solid lipid nanoparticles	Isoniazid	Inhibition of the synthesis of mycolic acid	TB	PP SA Poloxamer 188 Mannose Leucine	pH-sensitive response achieved by adding isoniazid as a prodrug	[118]
Liposomes	Liquorice extract (glycyrrhizin)	Membrane disruption	TB	Lipoid^®^ 100 Cholesterol	In total, 46% of the drug reached the lungs, with 16% remaining in the lungs 24 h post-administration	[119]
Nanogels	LLKKK18	Membrane disruption	TB	HA	Infection levels significantly reduced after just five to ten doses	[120]
Nanogels	Isoniazid Rifampicin	Inhibition of the synthesis of mycolic acid (isoniazid) + inhibition of RNA synthesis (rifampicin)	TB	Carboxymethyl chitosan Genipin	Selective targeting of lungs, maintaining drug concentrations of 40–60% after 24 h	[121]
Polymeric nanoparticles	Rifampicin Ascorbic acid	Inhibition of RNA synthesis (rifampicin) + adjuvant (ascorbic acid)	TB	Sodium alginate Tween^®^ 80 Sucrose Mannitol	Demonstrated activity against nine clinical strains of *M. tuberculosis*	[123]
Polymeric nanoparticles	Ciprofloxacin	Inhibition of type IV topoisomerase	Lower respiratory tract infections—unspecified	PEtOx Tannic acid	Sustained release over the course of 7 days	[127]
Polymeric nanoparticles	SET-M33	Binding to LPS	Infection caused by *P. aeruginosa*	Dextran	Prolonged residence time (12-fold higher) compared to free peptide; demonstrated efficacy and safety in vivo	[128]
Anionic liposomes	Levofloxacin	Inhibition of type IV topoisomerase	Infection caused by *P. aeruginosa* in cystic fibrosis patients	DSPC Cholesterol DSPE-PEG 2000	Sustained release over 72 h and demonstrated activity against five strains of *P. aeruginosa*	[129]
Liposomes	Levofloxacin Lysozyme	Inhibition of type IV topoisomerase (levofloxacin) + hydrolysis of peptidoglycan in bacterial cell wall (lysozyme)	Infection caused by *S. aureus*	Phospholipon^®^ 90G Phospholipon^®^ 90H Cholesterol Lactose	Demonstrated decrease in microbial burden in lungs, bronchoalveolar lavage fluid, and nasal fluid	[130]
Liposomes	Colistin	Displacement of magnesium and calcium in LPS	Infection caused by *P. aeruginosa*	Sodium cholesteryl sulphate Lipoid^®^ S75	Prolonged drug retention in the lung and enhanced in vivo efficacy	[131]

Key: tuberculosis (TB); palmityl palmitate (PP); stearyl amine (SA); hyaluronic acid (HA); poly(2-ethyl-2-oxazoline) (PEtOx); lipopolysaccharide (LPS); 1,2-distearoyl-sn-glycero-3-phosphocholine (DSPC); 1,2-distearoyl-sn-glycero-3-phosphoethanolamine-N-(polyethylene-glycol)-2000.

**Table 3:** Novel formulations to treat fungal pulmonary infections.

Formulation	Drugs	Mechanism of Action	Excipients	Comments	Reference
Polymeric nanoparticles	Voriconazole	Inhibition of ergosterol demethylation	Chitosan	Enhanced lung deposition and demonstrated in vitro efficacy against *Aspergillus*	[138]
Inorganic nanoparticles	Leaf extract of *Artemisia sieberi*	Not reported	Silver (AgNO_3_)	Demonstrated efficacy against *Aspergillus*, reducing lung tissue damage, and reduced oxidative stress	[139]
Liposomes	Dapsone	Inhibition of synthesis of dihydrofolic acid	DPPC Cholesterol	Prolonged in vitro release up to 16 h	[140]
Micelles	Amphotericin B	Membrane disruption	Chitosan Stearic acid	Aerosolisation of amphotericin B with improved activity compared to the free drug	[141]
Liposomes	Amphotericin B	Membrane disruption	Egg PC Cholesterol OPM OPP	Improved in vivo airway penetration in rats and accumulation in lung tissue for over 24 h	[143]
Nanostructured aggregates	Itraconazole	Inhibition of ergosterol demethylation	Mannitol Lecithin	Achieved lung deposition and systemic levels in mice	[144]

Key: 1,2-dipalmitoyl-*sn-*glycero-3-phosphocholine (DPPC), phosphatidylcholine (PC), O-palmitoylated mannan (OPM), O-palmitoylated pullulan (OPP).

### 3.3. Viral Infections

Respiratory infections caused by viruses typically impact the upper airways and are often self-limiting. However, certain infections can spread to the lower respiratory tract, developing into severe diseases, especially among children and the elderly in low- and middle-income countries [145]. Influenza and respiratory syncytial virus (RSV) are common viral agents responsible for significant morbidity in older adults. Recently, severe acute respiratory syndrome coronavirus 2 (SARS-CoV-2) had an unparalleled impact on healthcare systems and society.

Nanotechnology has emerged as a promising area for enhancing viral infection treatments [146] since nanocarriers can be engineered to target viruses at various stages of infection [147]. In particular, pulmonary-administered nanoformulations offer potential advantages for treating lung-related illnesses. Some examples of pulmonary formulations for viral infections are shown in Table 4.

#### 3.3.1. SARS-CoV-2

The virus responsible for coronavirus disease 2019 (COVID-19), SARS-CoV-2, was initially identified in Wuhan (China) in December 2019 and rapidly became a global pandemic [148,149]. COVID-19 has led to over 6 million deaths [150], with elderly individuals showing higher mortality rates [151]. To improve COVID-19 treatments, various pulmonary nanomedicines have been developed [152]. 

During the pandemic, remdesivir, a prodrug metabolically converted into a nucleoside triphosphate, received authorisation for emergency use from the U.S. Food and Drug Administration (FDA) for hospitalised patients with severe COVID-19 [153]. Remdesivir was among the first drugs to demonstrate improved patient outcomes; however, its use is limited due to its physicochemical properties and pharmacokinetics, including metabolism by both CYP and non-CYP enzymes and its incompatibility with oral administration. Additionally, it has systemic side effects, such as hepatotoxicity [154]. Pulmonary administration has been explored as a way to address these issues and enhance the efficacy of remdesivir.

Sanna and colleagues designed inhalable polymeric nanoparticles loaded with remdesivir to target the human angiotensin-converting enzyme 2 (ACE2), which SARS-CoV-2 utilises to infect host cells [155]. This formulation exhibited increased antiviral activity compared to free remdesivir [156]. Meng et al. demonstrated that dexamethasone-loaded nanoparticles, created with neutrophil-derived nanovesicles and cholesterol, reduced lung inflammation and injury in rhesus macaques infected with SARS-CoV-2. Notably, inhalation was more effective than intravenous administration, as the pulmonary delivery of 10 μg/kg dexamethasone outperformed intravenous administration of 100 μg/kg due to greater accumulation in inflamed lungs, as these nanovesicles showed improved targeting to macrophages, inhibiting the inflammatory response of a broad range of cytokines [157]. 

Fouad et al. developed an inhalable nanosuspension of remdesivir with polycaprolactone (PCL) and Pluronic^®^ F127, which was transformed into inhalable microparticles with hyaluronic acid and mannitol. This formulation showed improved drug release over free remdesivir, with a 1.5- and 1.9-fold increases at 24 and 48 h, respectively, along with an enhanced safety profile [158]. Halevas et al. created a nano-based delivery system using 2,2,-bis(hydroxymethyl)propionic acid (bis-MPA) dendritic nanocarriers to improve the solubility of remdesivir. Release studies indicated complete drug release within 24 h, following first-order kinetics. Toxicity tests on lung fibroblasts and alveolar macrophages confirmed non-toxic profiles similar to free remdesivir [159].

Ali et al. developed nanostructured lipid carriers for the pulmonary delivery of hydroxychloroquine, a repurposed drug during COVID-19. This drug presented a few limitations, including reduced efficacy and cardiac toxicity. The nanoformulation provided full drug release within 24 h and demonstrated effectiveness in reducing lung inflammation and injury in an acute lung injury mouse model. However, the cardiac toxicity of the formulation still needs to be assessed [160].

#### 3.3.2. Other Viral Infections

Nanomedicine-based approaches for viral infections extend beyond SARS-CoV-2. While the COVID-19 pandemic heightened focus on this virus, research has also targeted other respiratory viruses, including RSV and influenza [161]. Chang et al. developed resveratrol nanoparticles via sonication-assisted self-assembly, enhancing solubility and dissolution rates. Nebulised resveratrol nanoparticles improved pharmacological effects by inhibiting RSV replication and reducing pro-inflammatory cytokine production. RSV-infected mice treated with these nanoparticles showed reduced viral loads and improved lung microenvironment [161]. Peng et al. designed a chitosan-coated liposome delivery system for oxymatrine, a herbal compound with antiviral activity against RSV. Encapsulation within chitosan-coated liposomes enhanced lung tissue retention and improved therapeutic effects in RSV-infected mice by overcoming the mucous barrier [162].

Gil et al. created interferon-λ (IFN-λ)-loaded nanoparticles combined with pulmonary surfactants for inhalation against influenza A. This formulation notably restricted viral replication from day three post-infection and fully improved lung pathology, surpassing recombinant IFN-λ in effectiveness and immunological response, evidenced by increased monocyte frequency and restored T and B cell profiles [163].

**Table 4:** Novel formulations to treat viral pulmonary infections.

Formulation	Drugs	Mechanism of Action	Disease	Excipients	Comments	Reference
Polymeric nanoparticles	Remdesivir	Inhibition of RNA polymerase	COVID-19	PLGA PCL	Selective targeting to ACE2 membrane receptor and enhanced antiviral effect compared to free drug	[156]
Nanovesicles	Dexamethasone	Agonist of the glucocorticoid receptor	COVID-19	Nanovesicles were obtained from neutrophils from bone marrow of mice or rhesus macaques	Better outcome when the formulation was inhaled instead of injected. Improved targeting to macrophages.	[157]
Nanosuspension	Remdesivir	Inhibition of RNA polymerase	COVID-19	PCL Pluronic^®^ F127 HA Mannitol	Enhanced drug release compared to free drug (1.5-fold increase at 24 h and 1.9-fold increase at 48 h) and better safety index (1.3-fold higher).	[158]
Dendritic nanocarriers	Remdesivir	Inhibition of RNA polymerase	COVID-19	Hyperbranched G4-PEG6k-OH	First-order release kinetics with total release of remdesivir after 24 h and similar toxicity profile than free drug.	[159]
Nanostructured lipid carriers	Hydroxychloroquine	Inhibition of TLR9	COVID-19	Sweet almond oil Glyceryl behenate PC Gelucire^®^	Improved lung tissue targeting of hydroxychloroquine, but further investigation needed to confirm its potential for COVID-19 treatment	[160]
Nanoparticles	Resveratrol	Activation of Sirt-1	Infection caused by RSV	Unspecified	In vivo models demonstrated extended lung residence and reduced viral load compared to free drug	[161]
Liposomes	Oxymatrine	Antiviral activity induced by promoting difference cytokines	Infection caused by RSV	DPPC HSPC DPPG Cholesterol DSPE-PEG Chitosan	Selective distribution and improved retention in lung tissue compared to the free drug	[162]
Lipid nanoparticles	IFN-λ	Recruitment of neutrophils and NK cells	Influenza	Protamine Unspecified lipids Unspecified proteins	Improved delivery of IFN-λ to lungs and superior efficacy compared to recombinant IFN	[163]

Key: coronavirus disease 2019 (COVID-19); poly(lactic-co-glycolic) acid (PLGA); polycaprolactone (PCL); angiotensin-converting enzyme 2 (ACE2); hyaluronic acid (HA); toll-like receptor (TLR); phosphatidylcholine (PC); sirtuin-1 (Sirt-1); respiratory syncytial virus (RSV); interferon-λ (IFN-λ); natural killer cells (NK); dipalmitoylphosphatidylcholine (DPPC); hydrogenated soybean phosphatidylcoline (HSPC); dipalmitoylphosphatidylglycerol (DPPG); 1,2-distearoyl-*sn-*glycero-3phosphoethanolamine-*N*-[amino(polyethylene glycol)] (DSPE-PEG).

## 4. Nanovaccines

Nanostructures used in vaccine formulations can serve multiple purposes: they can act as adjuvants to enhance vaccine effectiveness, function as antigen delivery systems, or improve antigen presentation to the immune system or even serve as the primary component to the vaccine. The role of nanotechnology in vaccine development has a long history; for instance, two nanovaccines, Epaxal^®^ and Inflexal^®^, were approved in the 1990s for hepatitis A and influenza prevention, respectively [164]. These vaccines use virosomes (liposome-like particles embedded with viral proteins) as carriers [165]. Epaxal^®^ virosomes contain formalin-deactivated hepatitis A antigen within a phospholipid bilayer, facilitating absorption into immunocompetent cells [166,167], while Inflexal^®^ contains virosomal particles from three influenza strains, selected following WHO guidelines, that express hemagglutinin and neuraminidase glycoproteins [168].

The COVID-19 pandemic renewed interest in nanovaccine technology, with years of research enabling clinical trials to start rapidly after the genome of the virus became available [169]. Among approved COVID-19 vaccines, Spikevax (Moderna™) and Comirnaty (Pfizer Ltd./BioNTech SE) use lipid nanoparticles to stabilise mRNA and facilitate cellular uptake [169]. Spikevax includes lipids such as SM-102 (heptadecane-9-yl 8-((2-hydroxyethyl) [6-oxo-6-(undecyloxy)hexyl]amino)octanoate), 1,2-distearoyl-*sn*-glycero-3-phosphocholine (DSPC), cholesterol, and 1,2-dimyristoyl-rac-glycero-3-methoxypolyethylene glycol-2000 (PEG2000-DMG), while the lipid structure of Comirnaty incorporates ((4-hydroxybutyl)azanediyl)bis(hexane-6,1-diyl)bis(2-hexyldecanoate) (ALC-0315) and 2-[(polyethylene glycol)-2000]-*N*,*N*-ditetradecylacetamide (ALC-0159) for mRNA electrochemical interaction, with cholesterol and DSPC added to enhance stability [170,171]. Ultimately, PEG-modified lipids are incorporated to enhance the solubility of the lipid nanoparticles [172]. These PEGylated lipids, lacking an ionic charge, contribute to the colloidal stability of the lipid nanoparticles. Furthermore, the PEGylated lipids confer the ability for the vaccine to evade phagocytosis by the mononuclear phagocyte system [173]. 

Beyond the commonly used subcutaneous and intramuscular administration, the pulmonary delivery of nanovaccines has gained attention, especially for respiratory infections. This route is crucial for establishing mucosal immunity, which can prevent pathogen transmission by inducing tissue resident memory T cells and immunoglobulin A antibodies [174,175]. Subcutaneous or intramuscular administered vaccines promote systemic immunity but do not offer mucosal immunity. The vaccines must be administered by the nasal or pulmonary administration routes to develop robust mucosal immunity in the respiratory tract [176,177]. Nanocarriers explored for pulmonary delivery include liposomes, polymeric nanoparticles, lipid–polymer hybrids, and virus-like particles (VLPs). Several nanovaccines for respiratory diseases like COVID-19 and influenza have shown promise in this approach. A few examples of these nanovaccines for pulmonary delivery have been summarised in Table 5. 

For example, Wang et al. developed a hybrid nanovaccine by combining nanovesicles expressing the receptor-binding domain (RBD) with pulmonary surfactant-mimicking liposomes containing monophosphoryl-lipid-A (MPLA). This nanovaccine provided notable advantages over traditional subcutaneous vaccination, including efficient targeting and activation of alveolar macrophages via the toll-like receptor 4/nuclear factor κ-light-chain-enhancer of activated B cells (TLR4/NF-κB) pathway. This process induced strong T and B responses, yielding high levels of RBD-specific IgG and secretory IgA antibodies. Additionally, it demonstrated broad neutralising activity against several SARS-CoV-2 variants (wild type, delta, and omicron pseudoviruses) while showing reduced side effects, with this inhalable delivery system particularly enhancing mucosal immunity [178].

Zheng et al. designed a bionic-virus nanovaccine comprising polyionisic–polycytidylic acid (poly(I:C)) as an adjuvant, biomimetic pulmonary surfactant liposomes as a virus-like capsid, and SARS-CoV-2 RBDs as the spike protein. This nanovaccine, designed for intranasal or inhalable administration, closely mimicked natural SARS-CoV-2 infection and elicited stronger mucosal immunity than conventional intramuscular or subcutaneous vaccines. This was demonstrated by elevated SARS-CoV-2-specific secretory IgA levels in respiratory secretions, effectively neutralising the virus [179].

Lokugamage et al. applied a systematic cluster-based approach to develop lipid nanoparticles comprising various lipids, either neutral or cationic helper lipids, and PEG. They discovered that the PEG molar ratio and the type of helper lipid were critical factors: lower PEG ratios performed best with neutral helper lipids, while higher ratios suited cationic lipids. This optimised nanoparticle design effectively delivered mRNA encoding antibodies against influenza H1N1, protecting mice from lethal infection and performing better than previous lipid nanoparticle systems intended for systemic delivery [180].

Lopez et al. designed a polyanhydride nanoparticle-based vaccine platform aimed at broad protection against influenza A virus. Several H3N2-based nanovaccine versions were tested, which induced robust systemic and mucosal antibody responses along with enhanced lung-resident and systemic cellular immunity. These immune responses were correlated with protection against both homologous and heterosubtypic infections [181].

**Table 5:** Nanovaccine formulations for pulmonary administration.

Formulation	Infection	Excipients	Comments	Reference
Nanovesicles + liposomes	COVID-19	DPPC, DPPE, DPPG, Cholesterol	Demonstrated neutralisation of multiple coronavirus variants and effective at generating mucosal immunity	[178]
Liposomes	COVID-19	DPPC, DPPG, DPPE-PEG-COOH	Elicited stronger mucosal protective immunity compared to intramuscular or subcutaneous vaccination	[179]
Lipid nanoparticles	Influenza A	DMPE, DPSE, Cholesterol, DOPE, DSPC, DOTAP, DOTMA, DODA	All the mentioned lipids were tested, but the composition of the optimised composition is unspecified. Successfully delivered mRNA encoding antibodies against influenza A	[180]
Polymeric nanoparticles	Influenza A	CPTEG, CPH	Elicited robust systemic and mucosal humoral immune responses and enhanced systemic and lung-resident cellular immunity	[181]

Key: coronavirus disease 2019 (COVID-19); dipalmitoylphosphatidylcholine (DPPC); 1,2-bis(disphenylphosphino)ethane (DPPE); dipalmitoylphosphatidylglycerol (DPPG); polyethylene glycol (PEG); 1,2-dimyristoyl-sn-glycero-3-phosphoethanolamine-N-[methoxy(polyethyleneglycol)-2000] (DMPE); 1,2-distearoyl-sn-glycero-3-phosphoethanolamine-N-[methoxy(polyethyleneglycol)-2000] (DPSE); 1,2-dioleyl-sn-glycero-3-phosphoethanolamine (DOPE); 1,2-dioleyl-3-trimethylammonium-propane (DOTAP); 1,2-di-O-octadecenyl-3-trimethylammonium propane (DOTMA); dimethyldioctadecylammonium (DODA); 1,8-bis(p-carboxyphenoxy)-3,6-dioxoctane (CPTEG); 1,6-bis(p-carboxyphenoxy)hexane (CPH).

## 5. Pulmonary-Administered Nanomedicines to Combat Lung Cancer

Lung cancer is the most diagnosed carcinoma and the main leading cause of cancer-related death, with more than 2 million new cases and nearly 1.9 million deaths worldwide in 2022 [182]. This neoplasm can be classified into small-cell lung cancer (SCLC) and non-small-cell lung cancer (NSCLC). This latter represents about 85% of all lung carcinomas and can be subdivided into different histological subtypes, including adenocarcinoma, adenosquamous carcinoma, squamous cell carcinoma, and large-cell carcinoma. Adenocarcinoma is the most common subtype, accounting for 50% of NSCLCs, and represents a significant health problem, as the 5-year survival rate is low (≈15%) [183]. 

Different treatment strategies are currently available for patients with lung cancer, including surgery, radiotherapy, immunotherapy, and chemotherapy. Tumour resection in combination with radiotherapy, is the preferable treatment option. However, most patients are not suitable for surgery due to local invasion or distant metastases. In these patients, systemic therapies, chemotherapy, and immunotherapy are the primary treatment strategies [184]. Delivering these drugs by the pulmonary route offers certain advantages (Figure 2) for treating lung carcinoma [185]. In fact, several clinical trials have evaluated the efficacy of inhalable chemotherapy (Table 6). Lemarie and collaborators demonstrated that the pulmonary administration of gemcitabine was safe and exhibited minimal signs of systemic toxicity, as very low plasma gemcitabine levels were detected. Around 42% of the administered dose remained in the lungs. Regarding anticancer activity, a minor overall response was detected in one patient and stable disease was detected in four patients. A total of 11 patients were enrolled in this study [186]. Similar results in terms of toxicity were also appreciated in patients treated with inhaled doxorubicin or 5-fluorouracil; meagre amounts of these antineoplastics were detected in the bloodstream [187,188]. In terms of efficacy, it should be noted that the administration of 5-fluorouracil was effective. A satisfactory anticancer effect was detected in 60% of treated patients [188].

Interestingly, Zarogoudilis and collaborators compared the efficacy of intravenous and inhaled carboplatin in NSCLC patients. They demonstrated that the pulmonary administration of carboplatin was more effective than its intravenous administration. The patients receiving inhaled carboplatin showed a higher, although non-significant, survival rate (benefit of 25 days). Nevertheless, these patients exhibited a lower lung function measured as forced expiratory volume in one second [189]. 

The studies mentioned above indicate that the pulmonary administration of antineoplastics allows for its localisation in the respiratory system; a meagre amount of antineoplastics reaches the bloodstream, reducing the systemic toxicity of these drugs. However, patients may experience an impairment in pulmonary function related to the local toxicity of these drugs that must be evaluated. In this context, administering antineoplastics encapsulated into nanocarriers may decrease their local toxicity and increase their anticancer activity, as these systems can be engineered to deliver drugs selectively at the tumour site [192]. 

Currently, there are two nanoformulations approved for lung cancer treatment: (i) Genexol-PM, polymeric micelles encapsulating paclitaxel, approved for the treatment of NSCLC and breast cancer in Korea, and (ii) Abraxane^®^, albumin nanoparticles containing paclitaxel approved by FDA and EMA for the treatment of advanced NSCLC and metastatic breast and pancreatic carcinomas. EMA recently authorised a bioequivalent formulation of Abraxane^®^, known as Pazenir^®^ [193,194]. It should be noted that these formulations are approved for intravenous administration. However, the efficacy of some inhalable nanoparticles encapsulating anticancer agents has also been tested in lung cancer patients. For example, Wittgen and colleagues evaluated the efficacy of inhaled liposomes encapsulating cisplatin in NSCLC patients. This formulation was, in general, safe without detecting dose-limiting toxicity. Signs of haematologic toxicity, nephrotoxicity, ototoxicity, or neurotoxicity (effects commonly associated with this antineoplastic) were not detected. The most common adverse effects were gastrointestinal (nausea and vomiting) and pulmonary (dyspnoea, fatigue, and hoarseness) toxicities. More than 40% of the patients exhibited these adverse effects. Regarding efficacy, tumour regression was not appreciated in any patient, but around 70% exhibited a stable disease [190]. Pulmonary-administered 9-nitrocamptothecin-loaded-liposomes were also safe at doses lower than 26.6 μg/kg/day. In this case, the most common adverse effects were nausea, vomiting, cough, bronchial irritation, and fatigue. However, higher levels of antineoplastics were absorbed systemically compared to the other formulations. Finally, in terms of efficacy, stable disease was detected in three patients with primary lung cancer [191]. 

In addition to these clinically tested formulations, many other pulmonary delivery nanosystems have been designed and evaluated at the preclinical level for lung cancer treatment (Table 7). Curcumin, a phenolic constituent in Curcuma longa L., attains significant interest in treating cancer, including lung carcinoma [195]. Zhang and collaborators developed curcumin-loaded liposomes of around 95 nm intended for pulmonary administration using a dry powder inhaler. This formulation demonstrated a higher lung deposition than free curcumin and a selective cytotoxic effect against lung cancer cells. Moreover, studies in rats also demonstrated higher anticancer activity. Tumour nodes and lung bleeding decreased in liposome-treated rats compared to the free-curcumin-treated group [196]. 

Similar results were also found with paclitaxel-loaded liposomes. The intratracheal administration of this formulation showed a 26-fold higher lung accumulation of paclitaxel compared to the intravenous injection in the tail vein. These liposomes also reduced tumour growth in the lungs and prolonged the survival rate of the mice [197]. The delivery of paclitaxel via other lipid nanoparticles (specifically nanostructured lipid carriers) was also effective. Pulmonary administration of these nanomedicines allowed for the accumulation of paclitaxel in the lungs, limiting systemic exposure and reducing systemic toxicity [198]. Moreover, a higher anticancer effect than free paclitaxel was obtained [199].

Erlotinib is a tyrosine kinase inhibitor approved for treating NSCLC overexpressing epidermal growth factor receptors [200]. Szabová et al. developed pegylated liposomes loaded for the pulmonary administration of this drug. These liposomes were able to penetrate the lungs. While air jet nebulisers led to deeper penetration, vibrating mesh nebulisers showed a lower leakage of the administered dose, as around 98% of the encapsulated dose was effectively delivered into the lungs [201]. The good penetration of this liposomal formulation through the respiratory system can be attributed, among other aspects, to PEG coating. As aforementioned, the presence of PEG on the surface of nanoparticles improves their penetration through the mucous layer and their access to deep lung regions. The anticancer efficacy of this formulation was not evaluated. However, it is expected to show an increased anticancer effect compared to free erlotinib due to its local delivery into the lungs. 

Hu and colleagues developed solid lipid nanoparticles of around 225 nm for the pulmonary administration of doxorubicin. This inhalable formulation allows for the delivery of higher doses of doxorubicin within the lungs than the pulmonary administration of free doxorubicin. Doxorubicin-loaded solid lipid nanoparticles exhibited a respirable fraction of around 79%, and non-encapsulated doxorubicin showed a significantly lower value of around 60%. Moreover, the encapsulated drug was able to reach deeper regions of the lungs. Higher plasmatic levels (≈2-fold higher plasma area under the curve values) were observed in rats treated with nanoparticles compared to animals receiving the free drug [202]. 

Apart from lipid nanoparticles, inhalable polymeric nanosystems have also been designed. Sorafenib-loaded inhalable PLGA nanoparticles showed suitable aerosolization properties and promising in vivo anticancer activity in an NSCLC tumour model [203]. Quinacrine, also known as mepacrine, is an antimalarial drug that recently demonstrated anticancer properties [204]. PLGA nanoparticles loaded with this agent and coated with albumin were also developed. It showed excellent aerosolization properties and an improved in vitro anticancer activity in NSCLC compared to the free drug. A higher apoptosis induction was also detected [205]. It should be noted that this superior efficacy can be attributed not only to the higher accumulation of quinacrine in the lung due to its pulmonary administration but also to the incorporation of albumin on the surface of quinacrine-loaded nanoparticles. As described above, albumin nanoparticles tend to accumulate in NSCLC due to the presence of GP-60 receptors in tumoral blood vessels and the overexpression of SPARC proteins in the tumour.

One advantage of nanomedicines is the possibility of co-encapsulating several compounds simultaneously, allowing for simultaneous release into the tumour environment. For example, Kaur and colleagues developed lipid nanoparticles containing paclitaxel and doxorubicin. The studies reported that this inhalable nanoformulation showed superior in vitro anticancer activity against A549 cells and higher drug distribution in the lungs compared to non-encapsulated antineoplastics [206]. Saimi and collaborators developed niosomes containing gemcitabine and cisplatin. They showed excellent aerosolization properties (aerosol output was 96.22%). This formulation exhibited significantly lower toxicity against healthy lung cells (MRC5 cell line). While the combination of free gemcitabine and free cisplatin was very toxic (IC_50_ < 1.6 µg/mL), the niosomal formulation containing both drugs was weakly toxic (IC_50_ = 280 µg/mL). This is interesting, as a lower toxicity in the lung tissue could be expected with this nanoformulation. However, regarding efficacy, a cytotoxic effect was also appreciated in lung cancer cells. In A549 cells, the administration of free drugs showed an IC_50_ < 1.6 µg/mL and for niosomes an IC_50_ of around 46 µg/mL. This lower antiproliferative effect could be related to the controlled release of these drugs [207]. 

**Table 7:** Inhalable nanomedicines developed at the preclinical level for treating lung carcinoma.

Nanocarrier	Drug	Observations	Reference
Liposomes	Curcumin	Better aerosolization properties. Selective cytotoxicity against lung cancer cells compared to healthy lung cells. Higher in vivo anticancer activity.	[196]
	Pirfenidone	Good aerosolization performance More cytotoxic effect against A549 cells than non-encapsulated drug.	[208]
	Paclitaxel	Higher lung accumulation of paclitaxel compared to i.v. administration. Tumour reduction compared to non-treated animals. Higher survival rate compared to non-treated animals.	[197]
	Erlotinib	Good aerosolization performance using vibrating mesh nebulisers.	[201]
Niosomes	Gemcitabine and paclitaxel	Aerosol output of 96.2%. Lower toxicity in healthy lung cells (MRC5) compared to free drugs (IC_50_ = 280 µg/mL vs IC_50_ < 1.6 µg/mL). Lower cytotoxic activity in lung cancer (A529 cells) compared to free drugs (IC_50_ = 46 µg/mL vs IC_50_ < 1.6 µg/mL).	[207]
Nanostructured lipid particles	Paclitaxel	Higher distribution in the lungs of the pulmonary route compared to intravenous injection. No signs of systemic toxicity after pulmonary administration.	[198]
	Paclitaxel	Better lung accumulation compared to free paclitaxel. Higher anticancer activity than free paclitaxel.	[199]
	Paclitaxel and Doxorrubicin	Higher antiproliferative effect in A549 cells. Higher distribution in the lungs compared to non-encapsulated drugs.	[206]
Solid lipid nanoparticles	Doxorubicin	Higher deposition of administered doses compared to inhaled free doxorubicin. Reach deeper regions in the lungs. Higher plasmatic level of doxorubicin compared to the administration of inhaled free doxorubicin.	[202]
Polymeric nanoparticles	Quinacrine (mepacrine)	Nanoparticles incorporating albumin on their surface. Good aerosolization properties. Improved in vitro anticancer activity in NSCLC compared to the free drug. Higher apoptosis induction.	[205]
	Sorafenib	Appropriate aerosolization properties. Higher in vivo anticancer activity in NSCLC	[203]

Key: intravenous (iv); non-small cell lung cancer (NSCLC).

## 6. Challenges in Clinical Translation

Despite substantial progress in developing nanomedicines for pulmonary administration, a significant gap remains between preclinical research and clinical translation [209]. Cost presents a critical barrier to nanomedicine development. Manufacturing these advanced medicines is substantially more expensive than traditional pharmaceutical approaches, resulting in significantly higher selling prices and acquisition costs for healthcare institutions [210]. The commercialisation landscape is predominantly shaped by small and medium-sized enterprises, who bear the burden of navigating extensive and expensive validation processes and clinical trial requirements, while larger pharmaceutical companies maintain a ‘low profile’, showing low interest in these emerging technologies [210]. This economic challenge particularly impacts affordability in middle- and low-income countries, effectively limiting potential market implementation [211].

Regulatory frameworks struggle to keep pace with rapid technological advancements, creating a complex approval environment [212]. The absence of standardised protocols for characterising nanomedicine formulations further complicates research efforts, as different research groups employ varying analytical tools and testing methods. This inconsistency makes comprehensive comparative assessments difficult [213]. 

While the scientific literature often emphasises successful experimental outcomes and promising results, this approach can inadvertently introduce publication bias. By underreporting failures, setbacks, and null results, researchers risk presenting an incomplete and potentially overly optimistic view of the current state of nanomedicine.

Additional developmental challenges include scaling production from laboratory to industrial levels, ensuring formulation stability and appropriate shelf life, addressing potential nanomaterial toxicity and achieving consistent drug loading and release profiles. Complex interactions with biological systems, potential long-term tissue accumulation effects, immune system responses, and environmental implications of nanomaterials further complicate research and development efforts.

## 7. Conclusions

The pulmonary route of administration is of interest in treating respiratory infections and lung cancer, as it would allow for the localisation of drugs in the lungs (action site) and a reduction in their systemic exposure. Nevertheless, the efficient pulmonary delivery of drugs is challenged, as the respiratory system (mainly through mucociliary escalator) tends to eliminate inhaled particles. Moreover, the lungs have a large surface area, and drugs can be rapidly translocated into the bloodstream and distributed to other organs. Certainly, nanomedicine holds the potential to overcome the limitations of the pulmonary route. Of all the types of nanosystems, lipid nanocarriers, particularly liposomes, are the most investigated nanomedicines. To date, one formulation, Arikayce^®^, consisting of liposomes of amikacin, is currently approved. It should be noted that although, commonly, drugs administered via the pulmonary route show less toxicity than drugs administered through other systemic routes, like intravenous administration, severe toxicity can occur at the pulmonary level and impair the respiratory function of patients. This aspect is especially problematic in the case of the administration of antineoplastics, which show high toxicity. The administration of nanomedicines is safer than the administration of non-encapsulated drugs. However, they are not devoid of causing severe lung damage and must be deeply investigated. Inhalable nanovaccines have the potential to revolutionise the prophylaxis and therapy of respiratory infections and lung cancer due to the improved mucosal immunity achieved through this route, although investigation in this field is still limited. To sum up, inhalable nanomedicines show great potential for administering antimicrobials and antineoplastics in patients suffering respiratory tract infections and lung carcinoma and for immunising against microorganisms entering by this route.

## Figures and Tables

**Table 1:** Inhaled formulations available for treating pulmonary infections.

Formulation	Drug	Pharmaceutical Dosage Form	Indication
Tobi^®^ *	Tobramycin	Solution for inhalation	Cystic fibrosis patients infected with *Pseudomonas aeruginosa*
Betkis^®^ *	Tobramycin	Solution for inhalation	Cystic fibrosis patients infected with *P. aeruginosa*
Cayston^®^ *	Aztreonam	Solution for inhalation	Cystic fibrosis patients infected with *P. aeruginosa*
Tobi^®^ Podhaler *	Tobramycin	Dry powder inhaler	Cystic fibrosis patients infected with *P. aeruginosa*
Kitabis^®^ Pak *	Tobramycin	Solution for inhalation	Cystic fibrosis patients infected with *P. aeruginosa*
Arikayce^®^ *	Amikacin	Liposomal inhalation suspension	Nontuberculous mycobacterial lung disease
Opelconazole	Opelconazole	Dry powder inhaler	Aspergillosis
PUR1900	Itraconazole	Dry powder inhaler	Fungal pulmonary infections
Voriconazole	Voriconazole	Dry powder inhaler	Fungal pulmonary infections
Apulmiq	Ciprofloxacin	Liposomal inhalation suspension	Chronic lung infections with *P. aeruginosa*
MRT5005	Ciprofloxacin	Lipid nanoparticles	Cystic fibrosis

* Approved formulations.

**Table 6:** Inhaled antineoplastics evaluated in clinics.

Drug	Dose	Cancer Type	Reference
Gemcitabine	1–4 mg/kg	NSCLC	[186]
Doxorrubicin	0.4–9.4 mg/m^2^	Primary and metastases in the lungs	[187]
5-Fluorouracil	250 mg	NSCLC	[188]
Carboplatin (iv or inhaled) +Docetaxel (iv)	Docetaxel: 100 mg/m^2^ Carboplatin: AUC 5.5	NSCLC	[189]
Liposomal cisplatin	1.5–48 mg/m^2^	NSCLC	[190]
Liposomal 9-Nitrocamptothecin	6.7–26.6 µg/kg/day	Primary lung cancer Metastases in the lungs	[191]

Key: non-small cell lung cancer (NSCLC); intravenous (iv); area under the curve (AUC).
